# Successful Treatment with Pemetrexed, Carboplatin, and Bevacizumab for Platinum-Resistant Adenocarcinoma of the Lung

**DOI:** 10.1155/2012/821280

**Published:** 2012-06-20

**Authors:** Sae Wada, Nobukazu Fujimoto, Kenichi Gemba, Michiko Asano, Yasuko Fuchimoto, Katsuichiro Ono, Shinji Ozaki, Takumi Kishimoto

**Affiliations:** Department of Respiratory Medicine, Okayama Rosai Hospital, 1-10-25 Chikkomidorimachi, Minamiku, Okayama 7028055, Japan

## Abstract

We present two cases of relapsed adenocarcinoma of the lung: a 50-year-old male and a 67-year-old male. Both patients had previously been treated with platinum-containing systemic chemotherapy. In both cases, significant clinical efficacy was demonstrated with combination chemotherapy consisting of pemetrexed, carboplatin, and bevacizumab as salvage treatment. Adverse events were mild. This regimen might be a viable therapeutic option even after heavy treatment such as platinum-containing chemotherapy, especially for patients with preserved organ function and good performance status.

## 1. Introduction

 Bevacizumab is an antivascular endothelial growth factor (VEGF) monoclonal antibody developed from murine antibody. It targets the VEGF ligand and impedes tumor-associated angiogenesis in a preclinical model [1]. Its clinical antitumor activity has been recently demonstrated. Furthermore, two randomized phase III trials documented its efficacy and safety profile in nonsquamous, nonsmall cell lung cancer (NSCLC) [2, 3]. Bevacizumab was approved in Japan for the treatment of nonsquamous NSCLC in 2009. However, the eligibility of patients for this therapy and the best drug combination is still controversial. We present two cases of adenocarcinoma of the lung; both patients had previously been treated with platinum-containing systemic chemotherapy. Significant clinical activity was demonstrated in both cases with combination chemotherapy consisting of pemetrexed, carboplatin, and bevacizumab as salvage chemotherapy. 

## 2. Case Presentations

**Case 1**

**Figure 1:**
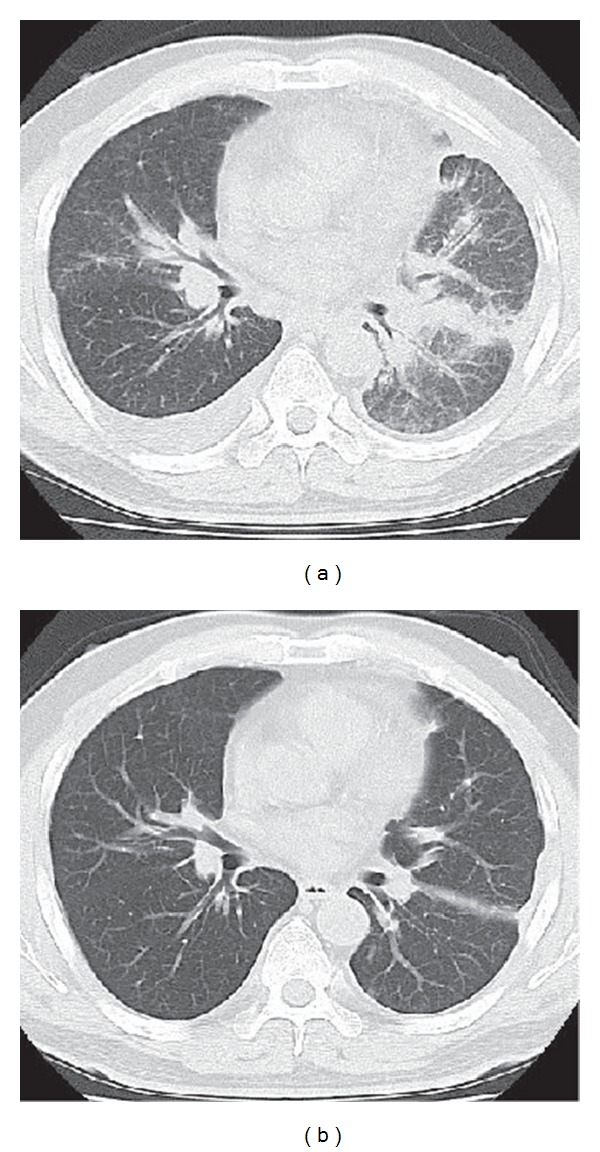
Chest CT of case 1 before chemotherapy demonstrating diffuse pleural dissemination of the tumor and irregular thickening of the bronchovascular bundle, indicating carcinomatous lymphangitis, and right pleural effusion (a). CT after two cycles of chemotherapy showing significant response (b).

A 50-year-old male was referred to our hospital with a dry cough and dyspnea on exertion. Chest radiograph demonstrated a left pleural effusion. Cytological examination of the fluid revealed adenocarcinoma of the lung. Computed tomographic (CT) scan of the chest after drainage of the pleural fluid revealed a tumor of the left lower lobe, diffuse pleural thickening, and mediastinal lymphadenopathy. The patient was ultimately diagnosed with lung adenocarcinoma (cT4N2M0, stage IIIB). After pleurodesis with OK-432, systemic chemotherapy consisting of cisplatin (80 mg/m^2^, day 1) and gemcitabine (1000 mg/m^2^, days 1 and 8) was initiated. Four cycles of therapy were administered with significant tumor shrinkage. Four months later, the patient again developed dyspnea on exertion, and CT scan revealed increased size of the pulmonary tumor and diffuse pleural dissemination with thickening of pleurovascular bundles, indicating carcinomatous lymphangitis (Figure 1(a)). Salvage chemotherapy consisting of pemetrexed (500 mg/m^2^, day 1), carboplatin (area under the curve 5, day 1), and bevacizumab (15 mg/kg, day 1) was administered. After two weeks, dyspnea on exertion was improved. Chest radiograph and CT demonstrated significant disease regression (Figure 1(b)). Therapy-related adverse events were mild and consisted of grade 3 neutropenia, grade 2 hypertension, and grade 1 anorexia. The patient was treated with an angiotensin-receptor antagonist for hypertension. Following four cycles of triplet chemotherapy, he continues to receive maintenance therapy consisting of bevacizumab and pemetrexed without relapse.

**Case 2**

**Figure 2:**
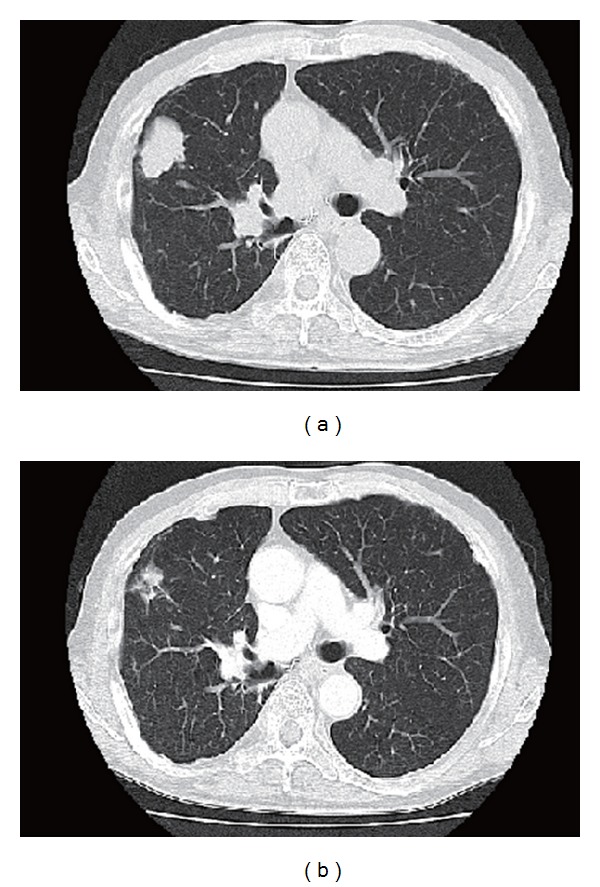
Chest CT of case 2 demonstrating tumor of the right upper lung (a). CT after the first cycle of the chemotherapy demonstrated remarkable response (b).

A 67-year-old male was diagnosed with lung adenocarcinoma in December 2001. He was an ex-smoker with a history of occupational asbestos exposure. The tumor was located in the right upper lung; he was also diagnosed with hilar and mediastinal lymph node involvement and bone metastasis to the right 5th rib (cT2N2M1, stage IV). Systemic chemotherapy consisting of cisplatin (60 mg/m^2^, day 1), docetaxel (60 mg/m^2^, day 1), and irinotecan (60 mg/m^2^, day 2) was initiated. Five cycles resulted in partial response. After chemotherapy, he underwent right upper lobectomy including resection of the 5th rib. In August 2004, the patient presented with metastasis to the thoracic vertebrae. He was treated with palliative radiotherapy and 4 cycles of salvage chemotherapy consisting of cisplatin (80 mg/m^2^, day 1) and docetaxel (60 mg/m^2^, day 1). Five months later, bone scan revealed multiple bone metastases including the lumbar vertebrae. Gefitinib was administered after palliative radiotherapy to the lumbar vertebrae, resulting in continued response for 5 years without any clinical symptom. Later tumor analysis revealed arginine-for-leucine substitution at amino acid 858 (L858R) in the EGFR kinase domain. In August 2010, CT scan revealed multiple pulmonary metastasis and right hilar lymphadenopathy (Figure 2(a)). Combination chemotherapy consisting of pemetrexed, carboplatin, and bevacizumab was administered at the same dosage and schedule as Case 1. Adverse effects were mild including grade 2 neutropenia and grade1 anorexia. CT after the first cycle of triplet chemotherapy demonstrated significant disease regression (Figure 2(b)). He has been in good physical condition with maintenance chemotherapy consisting of pemetrexed and bevacizumab.

## 3. Discussion

 Combination chemotherapy consisting of pemetrexed, carboplatin, and bevacizumab demonstrated significant clinical efficacy for previously treated adenocarcinoma of the lung in our patients. Both cases had been treated with platinum-containing regimens, and one of them had been heavily treated twice with platinum containing regimens and with gefitinib.

 For relapsed NSCLC, nonplatinum monochemotherapy is considered a standard treatment option. Pemetrexed, a multitargeted antifolate agent, is one of the standard treatment options based on a randomized clinical trial [4]. The addition of bevacizumab has not been established in Japan, but the combination of pemetrexed and bevacizumab demonstrated a favorable progression-free survival of 4 months when used as second-line therapy for patients with NSCLC in a recent report from the US [5]. The addition of a platinum agent to second-line treatment has not been established. However, Smit et al. [6] recently conducted a randomized phase II trial comparing pemetrexed with pemetrexed plus carboplatin in patients experiencing relapse after platinum-based first-line chemotherapy. They reported that pemetrexed plus carboplatin demonstrated favorable outcome in terms of disease-free survival compared to pemetrexed alone [6]. Their results suggest that platinum compounds plus pemetrexed may have a role in second-line treatment even in patients who received a platinum-based regimen. These data motivated us to deliver the combination chemotherapy to our patients with previously treated non-squamous NSCLC. The presented cases demonstrated significant clinical efficacy with mild adverse effects. The clinical activity of this regimen as the first-line treatment for elderly patients with non-squamous NSCLC was recently reported [7]. We think this regimen might be a viable treatment option even after heavy treatment such as platinum-containing chemotherapy, especially for relatively young patients with preserved organ function and good performance status. The clinical benefit of this combination chemotherapy should be confirmed in a large-scale clinical trial, particularly in comparison to pemetrexed alone or pemetrexed plus platinum.

 In conclusion, we present two cases of combination chemotherapy consisting of pemetrexed, carboplatin, and bevacizumab as salvage treatment showing significant clinical activity. The benefit of this triplet regimen as a first-line chemotherapy is under evaluation in a clinical trial. We propose that it should also be examined as salvage treatment.
